# Singlet Oxygen in Plants: Generation, Detection, and Signaling Roles

**DOI:** 10.3390/ijms21093237

**Published:** 2020-05-03

**Authors:** Valeriya A. Dmitrieva, Elena V. Tyutereva, Olga V. Voitsekhovskaja

**Affiliations:** Laboratory of Molecular and Ecological Physiology, Komarov Botanical Institute, Russian Academy of Sciences, Saint Petersburg 197376, Russia; VDmitrieva@binran.ru (V.A.D.); ETutereva@binran.ru (E.V.T.)

**Keywords:** acclimation, chloroplast, light stress, photosystem II, programmed cell death, retrograde- and plastid signaling, singlet oxygen

## Abstract

Singlet oxygen (^1^O_2_) refers to the lowest excited electronic state of molecular oxygen. It easily oxidizes biological molecules and, therefore, is cytotoxic. In plant cells, ^1^O_2_ is formed mostly in the light in thylakoid membranes by reaction centers of photosystem II. In high concentrations, ^1^O_2_ destroys membranes, proteins and DNA, inhibits protein synthesis in chloroplasts leading to photoinhibition of photosynthesis, and can result in cell death. However, ^1^O_2_ also acts as a signal relaying information from chloroplasts to the nucleus, regulating expression of nuclear genes. In spite of its extremely short lifetime, ^1^O_2_ can diffuse from the chloroplasts into the cytoplasm and the apoplast. As shown by recent studies, ^1^O_2_-activated signaling pathways depend not only on the levels but also on the sites of ^1^O_2_ production in chloroplasts, and can activate two types of responses, either acclimation to high light or programmed cell death. ^1^O_2_ can be produced in high amounts also in root cells during drought stress. This review summarizes recent advances in research on mechanisms and sites of ^1^O_2_ generation in plants, on ^1^O_2_-activated pathways of retrograde- and cellular signaling, and on the methods to study ^1^O_2_ production in plants.

## 1. Introduction

The most effective way to transform the energy of sunlight into chemical energy that has evolved naturally on Earth is oxygenic photosynthesis [1]. Its side-product is molecular oxygen in the ground triplet state (^3^O_2_), which is generated during oxidation of water coupled to the activity of photosystem II (PS II). Oxygenic photosynthesis thus enabled aerobic respiration necessary for most eukaryotic organisms populating the Earth. However, the appearance of oxygen in the atmosphere also increased the emergence of reactive oxygen species (ROS) which can be harmful, and even dangerous, for cells.

**Figure 1:**
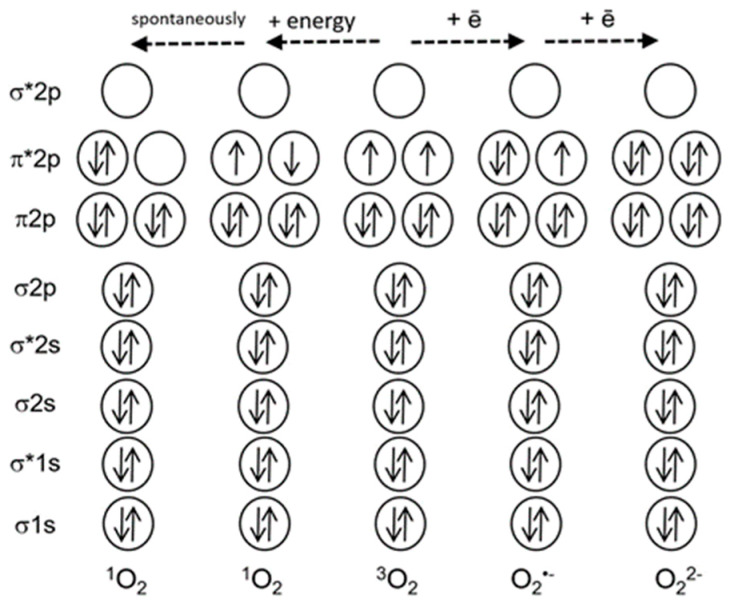
A simplified scheme of electron shells of the oxygen molecule and reactive oxygen species. Electronic orbitals are indicated, * marks antibonding orbitals. Solid arrows indicate electron spins (modified from [4]).

Figure 1 shows that oxygen in the ground triplet state (^3^O_2_) can be converted to ROS via two mechanisms, either via transfer of energy to an oxygen molecule, or via transfer of electrons. The first mechanism results in the formation of singlet oxygen (^1^O_2_), and the second mechanism leads to a sequential reduction of oxygen to the superoxide-anion-radical (O_2_^•−^), hydrogen peroxide (H_2_O_2_) and further to the hydroxyl radical (OH^•^) [2]. Other ROS, such as atomic oxygen or ozone, are not produced in living cells. ROS often damage cell components, like proteins, lipids and nucleic acids; at the same time, ROS are critical players in several processes, for instance, in plant protection from pathogens or in cell signaling [3].

Contrariwise to other ROS, ^1^O_2_ which represents the first excited electronic state of molecular oxygen is not a free radical, and does not carry a high-energy electron. Nevertheless, it easily oxidizes biological molecules and therefore can become toxic [5]. It also serves as a signal in plant cells and possibly in animal cells as well [6,7,8].

**Figure 2:**
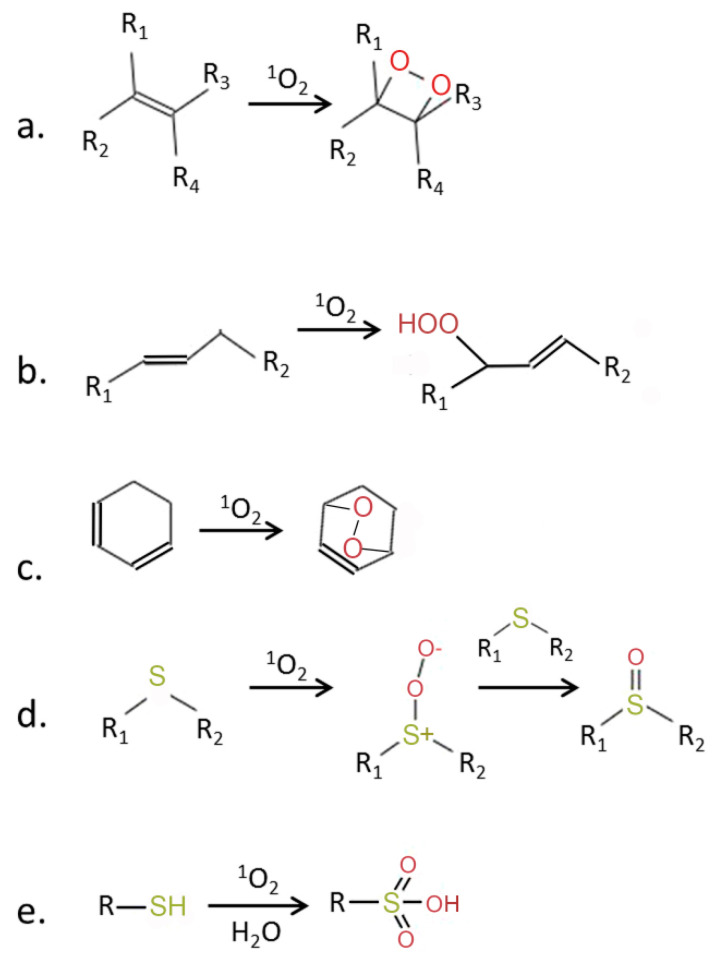
Chemical activity of ^1^O_2_ (modified from [5]). ^1^O_2_ is a strong electrophilic species which can react with molecules containing double bonds, leading to formation of (**a**) dioxitanes, (**b**) hydroperoxides, or (**c**) endoperoxides. Reaction with thiols leads to the oxidation of sulfur and formation of sulfoxides (**d**) and sulfonic acids (**e**). R, R_1_–R_4_—side chains.

Why is ^1^O_2_ much more reactive than oxygen in its ground state? The ground state of oxygen is a triplet, with two unpaired electrons with identical quantum spin numbers occupying different orbitals (Figure 1). Therefore, triplet oxygen can oxidize a non-radical (singlet) atom or molecule only when it contains a pair of electrons with parallel spins in free orbitals. However, electron pairs usually have opposite spin states. This limits the reactions of triplet oxygen with most organic molecules [9]. Singlet oxygen does not have a spin limitation (Figure 1), and this fact considerably increases its reactivity [9,10]. Figure 2 shows the main types of reactions involving ^1^O_2_.

In plant cells, ROS can be formed in many compartments including chloroplasts, mitochondria, peroxisomes and plasma membrane. Strong generation of ROS occurs in the light in chloroplasts during photosynthesis. Photosynthesizing leaves capture the energy of sunlight and use it to fix and reduce CO_2_. This is accompanied by the translocation of electrons through the electron transport chain (ETC) and by transfer of light energy between chlorophyll molecules, which occurs via generation of excited states of chlorophyll. Both processes can result in the formation of ROS by transfer of electrons and/or energy to oxygen.

ROS production in chloroplasts is especially high when light is available in excess; under these conditions, H_2_O_2_ and ^1^O_2_ are generated [9,11]. The largest part of hydrogen peroxide is formed during the dismutation reaction of O_2_^•−^ produced during the Mehler reaction via reduction of oxygen by electrons transferred from ferredoxin [12]. Small quantities of superoxide and hydrogen peroxide can be formed also in the lipid phase of thylakoid membrane in course of the reaction of oxygen with reduced plastoquinone [13]. A special role in ROS generation is played by photosystem II (PS II). The activity of PS II is driven by light energy captured via an ensemble of chlorophyll molecules and transferred to PS II reaction centers. PS II performs redox reactions with very high reducing, as well as oxidizing, redox potentials, including the unique reactions of water splitting. The latter process results in the release of oxygen, which is in its ground (triplet) state. However, PS II activity also leads to formation of ROS. The ROS which is predominantly generated at PS II is ^1^O_2_ [3,10]. This is due to the formation of long-lived excited triplet states of the P_680_ chlorophyll “special pair” in the PS II reaction center, which are able to transfer their excitation energy to oxygen, for instance, under conditions when electron acceptors downstream are reduced (see below) [11]. Interaction of oxygen with triplet chlorophyll (^3^Chl^*^) is the main pathway of ^1^O_2_ production in photosynthesizing organisms [3]. As PS II in chloroplasts is not only a site where light energy is transformed into chemical energy with occasional formation of ^3^Chl^*^, but also the site of oxygen production during photosynthesis, the close proximity of both processes further increases the probability of ROS generation at PS II.

## 2. Formation of ^1^O_2_ at PS II

Most ^1^O_2_ in plants is produced in the mesophyll cells of leaves. There, the main sources of ^3^Chl^*^ are light harvesting antenna complexes (LHC) and PS II reaction centers [5]. LHC contain the largest portion of leaf chlorophyll; however, as they also contain most of the carotenoids, about 95% of ^3^Chl^*^ formed in LHC is quenched [14]. This quenching occurs by a spin exchange leading to the formation of triplet carotenoids which easily dissipate the energy as heat [15]. Furthermore, under excess light a non-radiative mechanism of ^1^Chl^*^ inactivation, the energy-dependent non-photochemical quenching (qE), is activated in LHC which prevents the conversion of ^1^Chl^*^ to ^3^Chl^*^ by dissipation of energy as heat [5]. Nevertheless, LHC can generate ^1^O_2_ in vitro [16].

PS II contains β-carotene but the distance between chlorophyll and β-carotene molecules in PS II reaction centers is too large for quenching to occur. For triplet state quenching, the electron orbitals should overlap, and the distance between the molecules should not exceed 3.6 Å (van-der-Waals distance). In PS II, in contrast to LHC, this distance exceeds 3.6 Å [15]. ^1^O_2_ is the main ROS formed in PS II reaction centers even at low light intensities [17,18]; however, in high light, when the dissipation of excess light energy can no longer maintain the acceptor side of PS II in a partially oxidized state, the probability of ^1^O_2_ formation increases considerably [2]. 

**Figure 3:**
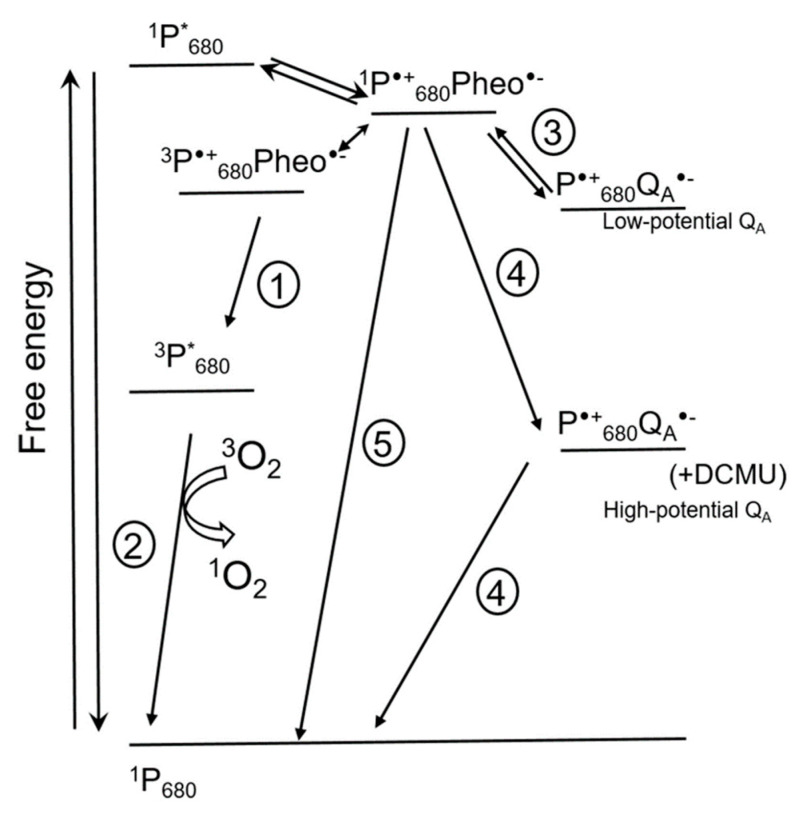
Scheme showing events in photosystem II (PS II) that can influence ^1^O_2_ formation (modified from [15]). The scheme shows direct recombination pathways of charge-separated state P^•+^_680_Pheo^•−^ leading to formation of ^3^P^*^_680_ and ^1^O_2_ (1 and 2), indirect recombination of P^•+^_680_Q_A_^•−^ involving repopulation of primary charge pair P^•+^_680_Pheo^•−^ (3), and non-radiative recombination leading to the formation of the ^1^P_680_ ground state (5). Under some conditions, for instance in the presence of 3-(3′, 4′-dichlorophenyl)-1, 1-dimethylurea (DCMU) [19], the potential of Q_A_^-^ increases, favoring non-radiative recombination of the P^•+^_680_Q_A_^•−^ pair, resulting in the formation of the ^1^P_680_ ground state (4). Double arrow shows spin conversion. * marks excited states. Pheo, pheophytin; Q_A_, plastoquinone A. For details, see text.

Figure 3 shows the pathways of ^1^O_2_ formation in PS II [15]. Chlorophyll molecules in LHC and in photosystems are in the ground singlet state. Chlorophyll excited singlet states have a lifetime long enough for conversion of excitation energy into the energy of electrochemical potential by means of charge separation in reaction centers of photosystems. This process occurs in chlorophylls of the ‘special pairs’ designated P_700_ for PS I and P_680_ for PS II, respectively, according to their spectral characteristics [18]. The first detected ion-radical pair formed in the reaction center of PS II after light excitation is the pair of a chlorophyll P_680_^•+^ and a pheophytin Pheo^•−^. If the transfer of an electron from Pheo^•−^ to the next acceptor is limited, charge recombination takes place, which can be accompanied by spin conversion and formation of a triplet excited state of ^3^P^*^_680_ with lower energy (1 in Figure 3). Triplet excited chlorophyll has a longer lifetime (10^−3^ s) than singlet excited chlorophyll (10^−8^ s), and can interact with oxygen and form ^1^O_2_ (2 in Figure 3). Thus, charge recombination in reaction centers of PS II leading to formation of ^3^P^*^_680_ promotes ^1^O_2_ formation [18]. Direct interaction of ^3^P^*^_680_ with triplet oxygen is supported, for instance, by the fact that addition of oxygen shortens the lifetime of ^3^P^*^_680_ from 1 ms to 30 µs; this also lowers the stability of PS II, presumably due to ^1^O_2_ formation [10]. 

The life span of oxidized P^•+^_680_ is very short due to a fast transfer of electrons from water to P^•+^_680_, but also due to a possibility of charge recombination of P^•+^_680_ with, apart from Pheo^•−^, other primary electron acceptors, Q_A_^•−^ and Q_B_^•−^ (3 in Figure 3), which sometimes leads to the formation of ^3^P^*^_680_ (1 in Figure 3). The probability of recombination increases under conditions when the ETC is over-reduced. Figure 3 shows that this can increase the rate of ^1^O_2_ formation via reactions 1 and 2. 

The redox potential of the plastoquinone Q_A_ in PS II can vary (Figure 3); for instance, it increases upon 3-(3′, 4′-dichlorophenyl)-1, 1-dimethylurea (DCMU) binding to PS II [10]. A positive shift of the Q_A_ potential also occurs under cold stress [19,20,21,22] and in high light when a strong acidification of the thylakoid lumen leads to Ca^2+^ release from the oxygen-evolving complex (OEC) of PS II [15,23]. These data were obtained not only with isolated thylakoid membranes but also in vivo. As Figure 3 shows, the probability of charge recombination between P^•+^_680_ and high-potential Q_A_^•−^ with formation of the P^•+^_680_Pheo^•−^ pair is very low; the probability of a non-radiative charge recombination to the ground states is much higher (4 in Figure 3). Accordingly, the probability of ^1^O_2_ formation decreases as well. Apart from this, high-potential Q_A_^•−^ can recombine with oxidized components of the OEC [15,23]. Thus, an increase in the potential of Q_A_ under stress protects PS II from photoinhibition by means of lowering ^1^O_2_ production while increasing the probability of “safe” charge recombination for the ion-radical pair P^•+^_680_Q_A_^•−^ [15]. Another ‘safety valve’ is a direct non-radiative charge recombination of P^•+^_680_Pheo^•−^ which results in the formation of ^1^P_680_ and Pheo (5 in Figure 3).

A positive shift of the Q_A_ potential also occurs in inactive reaction centers of PS II, where the Mn cluster within the OEC has been destroyed by light [24,25]. According to Johnson et al. [24], non-radiative recombination (4 in Figure 3) takes place in such complexes, lowering the probability of ^1^O_2_ formation. 

On the other hand, some stresses, for instance, drought, that cause stomata closure and a drop of the CO_2_ concentration in chloroplasts, lead to over-reduction of the ETC and enhance the probability of charge recombination between P^•+^_680_ and low-potential Q_A_^•−^ in PS II [15,26,27]. This leads to an increase in ^1^O_2_ production.

**Figure 4:**
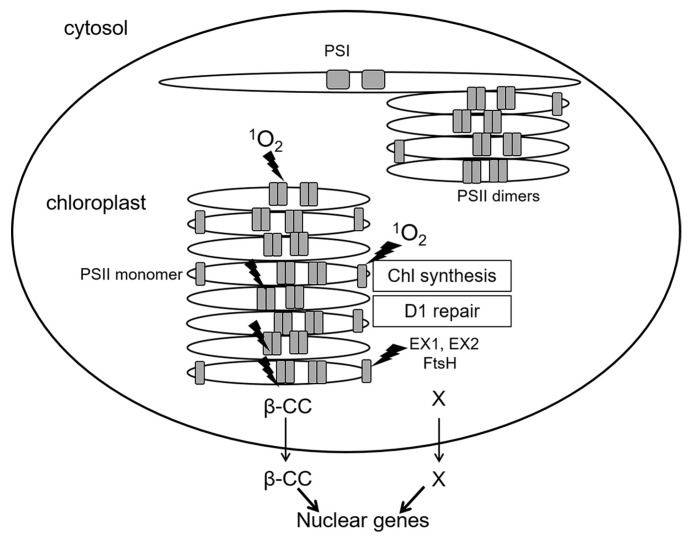
Spatial separation of the main processes of ^1^O_2_ generation in PS II. Shown are functionally active PS II complexes (dimers) in stacked parts of grana thylakoids (grana cores) as well as PS II monomers during repair or de novo assembly of PS II at grana margins, and also photosystem I (PS I) complexes in stromal thylakoids. Sites of ^1^O_2_ generation are designated by lightning symbols. Generation of ^1^O_2_ in grana cores and in grana margins, respectively, initiates signaling cascades transmitted outside chloroplasts via β-CC (for grana cores) and EX proteins (for grana margins). Thin arrows mark the exit from chloroplasts to the cytosol, thick arrows show an effect on nuclear gene expression. β-CC, beta-cyclocitral; EX1, EX2—EXECUTER1/2; FtsH, protease. X marks hypothetical products of the proteolysis of EX proteins by FtsH, which might represent plastid signals.

A special case is represented by inactive reaction centers of PS II which lack an OEC: immature PS II during de novo assembly, and damaged PS II in the course of repair [6]. While functional PS II complexes locate in stacked parts of chloroplast grana membranes (grana cores), de novo assembly and repair of PS II occur in grana margins (Figure 4). As mentioned above, in PS II reaction centers lacking OEC, the redox potential of Q_A_ is shifted to positive values, and the probability of ^1^O_2_ generation via formation of ^3^P^*^_680_ is negligible [23]. At the same time, PS II reaction centers are sites of ^1^O_2_ production not only in grana cores, where functionally active PS II complexes are situated, but also in grana margins [6]. Moreover, ^1^O_2_ can elicit contrasting signaling pathways in plant cells depending on the site of production—either grana cores or grana margins (see below). Thus far, the exact mechanism of ^1^O_2_ generation in grana margins remains elusive; supposedly, ^1^O_2_ can be produced by chlorophyll molecules during their incorporation into PS II complexes in the course of their repair or de novo assembly [6,25]. Both incomplete PS II complexes and enzymes of chlorophyll biosynthesis are located in unstacked marginal parts of the grana [28] (Figure 4). Chlorophyll molecules as well as their precursors can function as photosensitizers and lead to ROS production when not bound to proteins. To avoid this, chlorophyll precursors as well as products of chlorophyll catabolism are bound to special Light-harvesting CP-like proteins (LIL proteins) [25]. Nevertheless, production of ROS, especially ^1^O_2_, during assembly, repair and disassembly of PS II cannot be excluded [6].

In some studies, generation of ^1^O_2_ in vitro was demonstrated in isolated thylakoid membranes containing PS II complexes lacking an OEC [29,30]. In such complexes, the long-lived ion-radical pair P_680_^•+^TyrZ^•^ (where TyrZ is the tyrosine residue in the D1 protein which transfers the electrons from OEC to P_680_^•+^) was formed in the light which led to the generation of lipid and protein hydroperoxides [29,30]. Oxidation of these hydroperoxides led to the production of peroxyl-radicals and tetrahydroperoxides; the latter can decompose leading to the formation of triplet carbonyls able to perform energy transfer to ^3^O_2_ thereby producing ^1^O_2_ [29,30]. The importance of this mechanism in vivo has not been studied yet. Notably, in a similar study, no ^1^O_2_ generation was observed in thylakoid membranes enriched with PS II complexes lacking OEC [31]. To sum up, functional PS II reaction centers in grana cores generate ^1^O_2_ via charge recombination with the participation of low-potential forms of Q_A_^•−^ [15], while the mechanism of ^1^O_2_ formation at grana margins is not yet completely clarified [28].

## 3. Photoinhibition and the Role of ^1^O_2_

Photoinhibition is the phenomenon of down-regulation of photosynthesis, primarily of the rate of photosynthetic fixation of CO_2_, in the light caused by irreversible damage to PS II [32,33]. Of all components of the photosynthetic machinery, PS II has the shortest lifetime and the highest susceptibility to damage; moreover, plants might “sacrifice” PS II to protect PS I from over-reduction and photooxidation, as the repair of PS I is more complicated and energy-demanding than that of PS II [34,35]. Photoinhibition mostly occurs under high light; a term “midday depression of photosynthesis” has been related to this phenomenon [32]. A decrease in photosynthesis observed during long exposure of plants to high light can become more severe under limitation of photosynthetic electron transport. For instance, the reason for “midday depression” is the closure of stomata caused by increasing water loss around noon, which limits the concentration of CO_2_, and thereby of the final electron acceptor 1,3-diphosphoglycerate, and leads to over-reduction of the ETC [33].

During photosynthesis, two processes continuously occur: (1) the damage of PS II reaction centers, and (2) their repair. Photoinhibition is observed when process (1) occurs faster than process (2). The D1 protein, the core components of a PS II reaction center, is prone to the highest damage, although other PS II components can also be damaged, for instance, D2, CP43, and CP47 proteins [6]. In chloroplasts, D1 is the protein synthesized at the highest levels. Studies of photoinhibition using plants and cyanobacterial models led to two main hypotheses about the mechanism of D1 damage in the light. The first hypothesis postulates that the primary damaging agent is ^1^O_2_, production of which increases in excess light. The second hypothesis considers the Mn cluster of the OEC as the site of primary damage, which then leads to the formation of long-lived oxidized cation-radicals TyrZ^•+^ at the donor side of PS II, resulting in the oxidation of nearby amino acid residues of the D1 protein [6,36,37].

The decrease in photosynthesis during photoinhibition depends on the light intensity but not on the light spectrum [37]. In contrast, light-induced damage of D1 proteins is proportional to light intensity and to photon energy: UV and blue light cause the highest damage [36]. This action spectrum corresponds to the light spectrum causing damage to the OEC Mn cluster [36]. Obviously, as long as the rate of D1 biosynthesis is sufficient for the repair of PS II, there will be no photoinhibition. However, D1 biosynthesis can be inhibited in excess light, and this inhibition correlates with an increase in ^1^O_2_ generation [36]. In excess light, over-reduction of the ETC leads to an increase in ROS formation, primarily of ^1^O_2_, at PS II complexes (Figure 3) [6,37]. ^1^O_2_ attacks the histidine residues of PS II proteins, which can lead to modifications of PS II reaction centers and exposure of D1 to proteolytic degradation [31,38]. However, the strongest effect on the development of photoinhibition is related to the suppression of protein biosynthesis in chloroplasts by ^1^O_2_ which oxidizes cysteine residues of the elongation factors EF-G [36,39] and EF-Tu [40]. This suppression of protein biosynthesis in chloroplasts is fully reversible in that the oxidized cysteine residues can be reduced by thioredoxins [36].

These data were obtained using cyanobacterial models. Although also in plants, inhibition of protein translation in chloroplasts by increasing ^1^O_2_ production is considered the main factor of suppression of D1 biosynthesis, there is no direct evidence for oxidation of elongation factors by ^1^O_2_ in plants thus far [6]. As discussed above, biosynthesis of D1 protein takes place at grana margins, where the newly synthesized D1 binds chlorophylls and is incorporated into PS II reaction centers during their repair. The FtsH protease, which is also localized at grana margins [41], performs the specific proteolysis and removal of oxidized D1 from PS II [42].

Thus, photodamage of PS II is unavoidable due to the fact that even under low light conditions, light has a damaging effect on the OEC Mn cluster. However, occurrence and extent of photoinhibition depend on the rate of PS II repair, i.e., primarily on the rate of D1 biosynthesis in chloroplasts, which can be inhibited by ROS, mostly by ^1^O_2_ [37]. Protection from photoinhibition thus should include prevention of ^1^O_2_ production at PS II. Accordingly, DCMU has a slight protective effect (Figure 3; [43]). Photoinhibition in pumpkin plants was lowered by a strong magnetic field interfering with spin conversion in oxygen molecules and thus with ^1^O_2_ formation. This effect was not observed in the presence of lincomycin, an inhibitor of protein synthesis in chloroplasts, confirming that ^1^O_2_ inhibits PS II repair via an effect on protein synthesis [44].

## 4. Other Sites of ^1^O_2_ Generation in Plant Cells

^1^O_2_ is typically formed during the transfer of energy from an excited triplet chromophore molecule (a photosensitizer) to ^3^O_2_ [45,46]. Furthermore, a triplet-triplet transfer of energy from triplet carbonyls of lipids and proteins, ^3^C = O^*^, to ^3^O_2_ can occur [30]. ^1^O_2_ can form also during decomposition of tetrahydroperoxides of lipids and proteins, C-OOOO-C, via a mechanism proposed by Russell [47]. 

In chloroplasts, not only chlorophylls but also chlorophyll precursors such as chlorophyllide and protochlorophyllide, as well as chlorophyll catabolites, can function as photosensitizers and lead to ^1^O_2_ generation [10,25]. However, ^1^O_2_ is not produced at PS I [5]. When oxidized, the “special pair” chlorophylls of the PS I reaction centers, P^+^_700_, do not perform recombination reactions. Under reducing conditions when the Fe-S clusters of PS I are reduced or phylloquinon is removed from the reaction center, charge recombination can occur and lead to formation of ^3^P_700_ at room temperature [48]. However, the lifetime of ^3^P_700_ in PS I is only about 6 µs (compared to 1 ms for ^3^P_680_), and it does not decrease upon addition of ^3^O_2_, suggesting that P_700_ is shielded from O_2_ [10,49]. Interestingly, an *Arabidopsis* mutant with low contents of β-carotene showed photoinhibition of PS I and formation of ^1^O_2_ in LHC of PS I [50]. In wild-type plants, photoinhibition of PS I can be induced by multiple saturating light pulses; this was suggested to result in the generation of not only O_2_^•−^ and H_2_O_2_, but also ^1^O_2_ at PS I [51]. Altogether, more evidence is required to support this suggestion.

The cytochrome-b6f complex can produce ^1^O_2_ in the light [52]. Here, the Fe-S cluster of Rieske proteins, as well as a chlorophyll with unknown functions, are probably the sites of ^1^O_2_ generation [10].

^1^O_2_ can also be produced in cell compartments other than chloroplasts in a range of enzymatic reactions including those of heme proteins and lipoxygenases [53]. Other sources of ^1^O_2_ in plants can be phytoalexins, defense compounds that are synthesized upon pathogen attack and can accumulate in every plant organ. Phenalenone-like phytoalexins are photosensitizers, which function as phototoxins generating ^1^O_2_ to kill pathogens [54]. Phenalenone can generate ^1^O_2_ with a quantum yield around 1, which explains the fungicidal activity of phenyl-phenalenone phytoalexins [54]. Some plants constitutively produce phytoanticipins, secondary metabolites with antimicrobial activity [55], some of which can function as photosensitizers. For instance, psoralenes and a polycyclic quinone hypericin are phototoxins of the *Apiaceae* family and of the genus *Hypericum*, respectively [5]. In animal cells, ^1^O_2_ can be generated in neutrophils during the myeloperoxidase reaction in the course of phagocytosis [56]; thus far, it is not known whether similar reactions play a role in plants’ defense against pathogens. At any rate, in *Arabidopsis thaliana*, chlorophyll catabolites can be used as photosensitizers during the hypersensitivity response against the pathogen *Pseudomonas syringae* [57].

^1^O_2_ can be produced also in underground plant organs in the absence of light [58,59]. In roots of *Arabidopsis thaliana*, osmotic stress induced by polyethylene glycol (PEG) elicited ^1^O_2_ production in the rhizodermis of the root tip, and later in the root apical meristems. This was accompanied by the death of root meristem cells and an increase in lateral root formation. In roots subjected to osmotic stress, the source of ^1^O_2_ could be hydroperoxides of fatty acids produced by lipoxygenase from linoleic acid; these hydroperoxides can form tetrahydroperoxides which decompose with generation of ^1^O_2_ according to Russell’s mechanism [47,60,61]. Apart from lipoxygenase reaction, hydroperoxides of linoleic acid in roots could be formed in reactions with hydroxyperoxyl radicals or hydroxyl radicals [4,59].

In tobacco Bright Yellow 2 (BY-2) suspension culture cells, ^1^O_2_ was produced in the dark in the apoplast in response to hyperosmotic stress induced by salt or sorbitol, supposedly during peroxidase reactions in cell walls [62,63,64]. The rapid production of ^1^O_2_ induced Ca^2+^ entry in tobacco cells and activated the SOS (Salt Overly Sensitive) signaling cascade necessary to protect cells from accumulation of excess Na^+^ and development of programmed cell death (PCD; [65]).

## 5. Damaging Effects of ^1^O_2_

Although ^1^O_2_ is metastable and its lifetime in the cells is short, a portion of ^1^O_2_ can diffuse over considerable distances both in polar and non-polar environments [15,17]. The lifetime of ^1^O_2_ can differ by several orders in various organic solvents [66]. In cell culture, ^1^O_2_ lifetime is about 10^−6^ s, and it can diffuse to a distance of ca. 155 nm [67] and permeate through membranes. In tissues, ^1^O_2_ rapidly attacks neighboring biomolecules such as DNA, pigments, lipids and proteins, causing oxidative damage [11,68]. 

In cells, ^1^O_2_ rapidly oxidizes molecules containing C-C double bonds, forming hydroperoxides or endoperoxides [27]. The main targets of ^1^O_2_ are double bonds of aromatic amino acid residues in proteins, polyunsaturated fatty acids, guanine bases in nucleic acids and thiols groups (Figure 2). These hydroperoxides can initiate free radical chain reactions, e.g., upon interaction with light or with hydroxyl radicals, which multiplies the damaging effects of ^1^O_2_. 

The most common targets of ^1^O_2_ are proteins [45]. Generally, proteins are the main targets of oxidative damage in cells due to their large amounts and high reactivity with radicals and excited molecules including ^1^O_2_. Reactions of tyrosine, tryptophan and histidine, both free and as part of proteins, with ^1^O_2_ result in high levels of hydroperoxide formation [69]. In proteins, ^1^O_2_ rapidly oxidizes these amino acids residues and also methionine, cysteine and phenylalanine. As noted above, during photoinhibition, ^1^O_2_ leads to a degradation of the D1 protein which plays a vital role in primary charge separation and stabilization of PS II [31,38], and also inhibits protein translation in chloroplasts, thus inhibiting D1 repair [36]. 

Interaction of ^1^O_2_ with lipids induces their rapid peroxidation [27]. ^1^O_2_-mediated lipid peroxidation can directly precede cell death [70]. The main target of ^1^O_2_ is linolenic acid, which leads to the generation of lipid hydroperoxides LOOH, such as 10- and 12-hydroperoxy-octadecadienic acids (HPODE) which represent reactive electrophile species [25]. In the presence of photosensitizers, lipid hydroperoxides can initiate free-radical reactions; therefore, they are detoxified by peroxidases forming other electrophile species such as 13-keto-octadecadienic and -trienic acids. These compounds can serve as signals: for instance, in the green alga *Chlamydomonas rheinhardtii*, many ^1^O_2_-activated genes contain «electrophile response elements» in their promoters and are induced by electrophilic lipids [71].

^1^O_2_ can interact also with DNA by inducing breaks in DNA strands and causing chromosome defects [72]. It reacts preferentially with guanine bases, forming 8-oxo-7,8-dihydroguanine and spiro-iminodihydantoin [73]. These products cause point mutations [25]. However, although ROS can cause severe damage in living cells, increasing amounts of data suggest that ROS-related cell death occurs mostly via activation of signaling pathways leading to PCD while cell death due to oxidative injuries is a relatively rare phenomenon [74].

## 6. Plant Defense Against ^1^O_2_

Quenching of ^1^O_2_ can occur by means of energy dissipation as heat without modifications of the quencher (physical quenching), leading to the formation of a ground state of ^3^O_2_ molecule, or by means of chemical quenching, leading to the formation of oxidized molecules [5]. In plant cells, carotenoids, tocopherols, plastoquinols and ascorbic acid are the main antioxidants protecting the cells from ^1^O_2_ [25].

As discussed above, ^3^Chl^*^ and ^1^O_2_ produced in LHC can be quenched very efficiently by carotenoids via the physical mechanism [5,18]. Although in PS II reaction centers, β-carotene cannot quench ^1^O_2_ via production of triplet states, it still protects PS II from ^1^O_2_ very efficiently by means of chemical quenching [75]. The products of this reaction are specific aldehydes and endoperoxides of β-carotene: β-cyclocitral, dihydroactinidiolide, and b-ionone [75]. These substances initiate signaling pathways, and their generation greatly increases in high light [5,25,76].

Tocopherols can also quench ^1^O_2_, but their rate of quenching is two orders of magnitude lower than that of β-carotene. α-Tocopherol can be oxidized by ^1^O_2_ to 8-hydroperoxytocopherol and further to α-tocopherolquinone, at this point, the pathway of α-tocopherol regeneration is unknown. Tocopherols also inhibit ^1^O_2_-initiated lipid peroxidation in thylakoids by reacting with lipid radicals, thus breaking free-radical reaction chains [77]. Data obtained for *C. rheinhardtii* showed that plastoquinol in thylakoids can be more effective in ^1^O_2_ quenching than tocopherols [78]. Moreover, chloroplasts contain high amounts of ascorbic acid [74] which also quenches ^1^O_2_ [79].

Tryptophan can quench ^1^O_2_ by a physical mechanism, similar to carotenoids, due to its chemical structure. More often, however, interaction of ^1^O_2_ with tryptophan leads to the production of peroxides via chemical quenching. These peroxides further can form stable compounds, such as N-formylkynurenine which can be used for identifications of the sites of ROS production [69]. Histidine, cysteine, methionine, tyrosine, and phenylalanine can also quench ^1^O_2_. The D1 protein is probably oxidized by ^1^O_2_ at the highest rate of all cellular proteins, and has a high rate of re-synthesis; therefore, it can be considered as a specific chemical quencher of ^1^O_2_ [18].

As reactions of ^1^O_2_ with cell components leads to the production of peroxides, reactive electrophilic species and other highly reactive molecules, diverse protective antioxidant systems are required for defense against ^1^O_2_: They can detoxify the reactive products and break free-radical chain reactions [9,74]. 

## 7. Roles of ^1^O_2_ in Plant Stress Response

While all ROS cause similar cytotoxic effects, the signaling pathways they activate are highly specific. Studies on the effects of ^1^O_2_ on plants are challenged by the fact that in the light, several types of ROS are produced simultaneously [80]. A very valuable tool for these studies are mutants that specifically produce high amounts of ^1^O_2_. One of them is the *flu* (*fluorescent in blue light*) mutant of *Arabidopsis thaliana* [81]. The nuclear-encoded chloroplast protein FLU regulates chlorophyll biosynthesis by preventing excess accumulation of the intermediates of Mg-protoporphyrin branch of tetrapyrrole synthesis. FLU forms a complex with proteins catalyzing final steps of chlorophyll synthesis, Mg^2+^-protoporphyrin IX monomethyl ester cyclase and NADPH:protochlorophyllide oxidoreductases B and C [82]. This complex localizes to grana margins [28]. Upon binding free protochlorophyllide, the complex inhibits glutamyl-tRNA-synthetase by blocking protochlorophyllide synthesis. The absence of FLU prevents formation of the glutamyl-tRNA-inhibiting complex, which causes accumulation of free protochlorophyllide in the dark [82].

The *flu* mutant remains viable when grown under continuous light: It shows growth pattern and seed production similar to those of wild type [83]. However, in the dark *flu* plants accumulate free protochlorophyllide. After transfer of *flu* plants from the darkness to light, protochlorophyllide acts as a strong photosensitizer leading to ^1^O_2_ generation [70,83]. Thus, the *flu* mutant represents a unique tool for “quantification” of ^1^O_2_ effects on plants, because the amounts of generated ^1^O_2_ are proportional to the amounts of accumulated protochlorophyllide, and the latter depends on the length of the dark period. By varying the time of incubation in darkness before transfer of *flu* plants in the light, dose effects of ^1^O_2_ on plants can be studied [84].

^1^O_2_ formation leads to death of *flu* etiolated seedlings, and to growth arrest in *flu* green plants [18,70,83]. Notably, neither death nor growth arrest in *flu* result from oxidative damage exerted by ^1^O_2_ but occur due to the activation of genetic programs of stress response [18]. ^1^O_2_-activated genes of early stress response differ from the genes activated by O_2_^•-^ and H_2_O_2_ [83]. Of special interest are genes that are specifically activated by ^1^O_2_ and function in PCD. In *flu* plants, two nuclear-encoded chloroplast proteins, EXECUTER1 (EX1) and EX2, trigger ^1^O_2_-dependent PCD [80]. In sharp contrast to *flu*, double mutants *flu/ex1* survive dark-to-light transfer even though their levels of accumulated protochlorophyllide and ^1^O_2_ production are similar to *flu* [18,85]. However, under severe light stress, even *ex1*/*ex2* plants die due to oxidative damage [86]. EX1 and EX2 proteins localize to grana margins [28]. An increase in ^1^O_2_ production leads to oxidation of these proteins triggering their proteolysis by the FtsH protease [28]. This proteolysis is related to the oxidation of the Trp643 residue in the DUF3506 domain of EX1; the DUF3506 is highly prone to oxidation by ^1^O_2_ and was designated SOS (singlet oxygen sensor) domain [87]. It is assumed that EX1 proteins can only be oxidized by ^1^O_2_ produced at grana margins; therefore, EX1/EX2-mediated signaling represents a ^1^O_2_-specific retrograde signaling pathway related to synthesis and/or repair of PS II [28].

The *Arabidopsis* EX1 protein per se mediates ^1^O_2_-dependent retrograde and/or plastidic signaling. It is degraded by FtsH, and presumably, products of its proteolysis function as signal. Notably, only levels of ^1^O_2_ sufficient to induce EX1 proteolysis can trigger PCD [28]. In the course of ^1^O_2_- and EX1/EX2-mediated PCD, levels of salicylic acid (SA) increase and ENHANCED DISEASE SUSCEPTIBILITY1 (EDS1) protein is activated; this protein is a key component of cell death during hypersensitive response elicited by biotrophic pathogens [88]. Accordingly, ^1^O_2_-dependent PCD is inhibited in *flu*/*eds1* double mutants [88]. The levels of jasmonates (JA) also increase in response to ^1^O_2_ production in the *flu* mutant [85,88]. In *Arabidopsis*, development of ^1^O_2_-dependent PCD also requires the presence of blue light receptor CRYPTOCHROME 1 (CRY1; [89]). Interestingly, development of PCD due to overaccumulation of protochlorophyllide was observed also in barley mutant *tigrina-d.1* orthologous to *Arabidopsis flu* mutant [90].

Recently, a novel chloroplast protein, SAFEGUARD1 (SAFE1), was shown to localize to the stroma and suppress EX1-dependent PCD in *Arabidopsis* [91]. SAFE1 protects grana margins from ^1^O_2_ and is degraded via chloroplast-originating vesicles when ^1^O_2_ levels rise. Interestingly, SAFE1 was also proposed to function in a novel retrograde signaling pathway leading to PCD induction independently of EX1 [91].

The *Arabidopsis ch*1 mutant represents another useful model to study ^1^O_2_ effects on plants. The *ch*1 plants lack chlorophyll *b* due to a mutation in the unique gene encoding chlorophyllide-*a*-oxygenase. In *ch*1 plants, the LHC antenna of PS II is strongly reduced which disturbs the functions of PS II and the processes of lateral diffusion in thylakoid membranes [80,92,93]. As a result, production of ^1^O_2_ in PS II reaction centers is strongly increased in *ch*1, without effects on the levels of other ROS [80]. However, in contrast to *flu*, ^1^O_2_ production at PS II in *ch*1 occurs in chloroplasts in stacked membranes of grana cores [80], and not in grana margins or in etioplasts as in *flu* [28]. 

Gene expression profiles in leaves of *ch*1 and *flu* after light stress showed high similarity: of more than 2600 genes with changed expression profiles in *ch*1, 80% showed a similar response in *flu* [80]. However, in contrast to *flu*, in *ch*1 EX1 does not participate in the onset of PCD [80]. In *ch*1, ^1^O_2_-dependent PCD involves a serine-threonine kinase OXIDATIVE SIGNAL INDUCIBLE1 (OXI1), which was previously shown to regulate H_2_O_2_-mediated processes including those related to the response to pathogens [94,95]. In a *ch1*/*oxi1* double mutant, high light leads to ^1^O_2_ generation but not to PCD [94]. OXI1 signaling involves phytohormones: expression of genes encoding enzymes involved in JA and SA biosynthesis is strongly increased in plants overexpressing *OXI1* (*OE-OXI1*) even in the absence of light stress, while in an *oxi1* knockout mutant the levels of SA, but not JA, were decreased [95]. Only a small number of genes is activated both during OXI-mediated and during EX1-mediated cell death [96]. Other genes induced in the *ch*1 mutant by high light are regulated by accumulation of hydroxyl-octadecatrienic acids due to ^1^O_2_-triggered lipid peroxidation [80].

Thus, ^1^O_2_ produced in stacked membranes of grana cores induces a specific type of cell death, namely OXI1-dependent PCD, while ^1^O_2_ produced at grana margins triggers EX1-dependent PCD. Both types of PCD depend on an increase in JA biosynthesis, which in turn activates the biosynthesis of SA triggering PCD. Importantly, these pathways differ from those activated during plant defense: Upon attack of necrotrophic pathogens, JA suppresses cell death induced by the release of O_2_^•−^ and H_2_O_2_. However, JA induces ^1^O_2_-dependent PCD during light stress. Although these data were obtained for *flu* and *ch*1 mutants, the corresponding signaling pathways were shown to function also in *Arabidopsis* wild-type plants. In extreme stress, for instance, when very high illumination is combined with cold temperature, wild-type plants show PCD mediated by OXI1 kinase and not by EX proteins [94]. 

Notably, exposure of *ch*1 plants to moderate light intensities before their transfer to high light prevents induction of *OXI* and the development of PCD [80,94]. Thus, *ch*1 plants can become acclimated to high light stepwise. The key component of the acclimation was shown to be an endoperoxide of β-carotene, β-cyclocitral, which is produced in PS II during an increase in ^1^O_2_ levels. This process might include also other ^1^O_2_-specific products of the oxidation of β-carotene, for instance, dihydroactinidiolide [75,76]. The acclimation includes the activation of genes encoding regulators of biosynthesis of plastoquinone and ubiquinone, anthocyanin and flavonoids, pyridoxine and methionine, and of other compounds which show a high reactivity with ^1^O_2_ [80]. Acclimation also leads to a decrease in expression levels of the genes encoding proteins related to mechanical damage as well as some transcription factors and proteins related to photosynthesis [80]. 

Of special interest is the inhibition of the expression of genes encoding enzymes involved in JA synthesis during the acclimation of *ch*1 to high light, as it contrasts sharply with the high expression levels of these genes in leaves of *ch*1 in high light. Acclimation was shown to involve the transcriptional regulators METHYLENE BLUE SENSITIVITY 1 (MBS1) [76] and SCARECROW LIKE14 (SCL14) [97], which are responsible for the inhibition of transcription of genes of JA synthesis and the induction of genes regulating detoxification processes, respectively. Both MBS1 and SCL14 are induced by β-cyclocitral, which is a sensor of light excess and of the increase in charge recombination in PS II. Interestingly, β-cyclocitral induces biosynthesis of SA during acclimation, but this does not lead to PCD [98].

**Figure 5:**
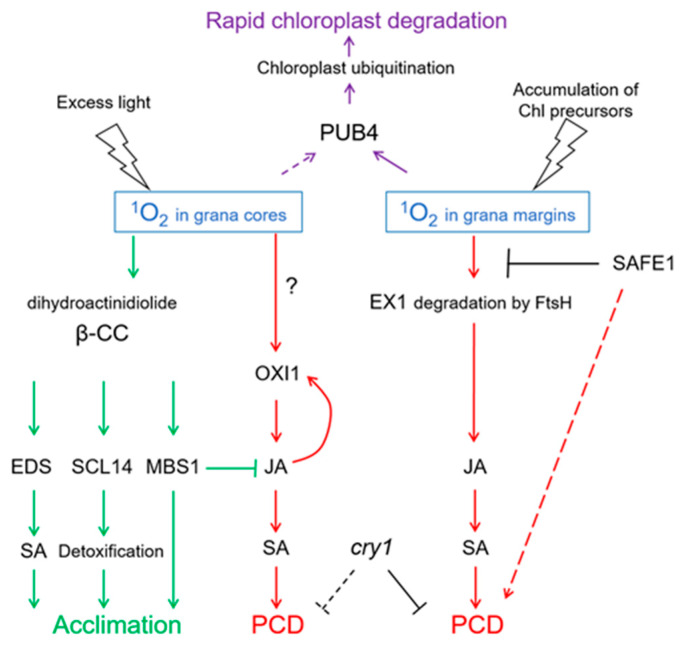
^1^O_2_-activated signaling pathways. Depending on the site of generation—in grana cores or in grana margins—^1^O_2_ induces programmed cell death (PCD) involving either OXIDATIVE SIGNAL INDUCIBLE1 (OXI1) kinase or EXECUTER1 (EX1) protein, respectively. Both types of PCD depend on the accumulation of salicylic acid (SA) induced by jasmonic acid (JA), and on the presence of the photoreceptor CRY1. SAFEGUARD1 (SAFE1) protein is located in the stroma and protects grana margins from ^1^O_2_, and in this way interferes with EX1-induced PCD. ^1^O_2_ generation in grana cores leads to oxidation of β-carotene within PS II, yielding β-cyclocitral (β-CC) and dihydroactinidiolide. These molecules activate the synthesis of SA, SCARECROW-LIKE 14 (SCL14)-mediated detoxification, and expression of acclimation-related genes depending on METHYLENE BLUE SENSITIVE1 (MBS). Altogether, this leads to acclimation to ^1^O_2_. Chloroplasts showing high levels of ^1^O_2_ production will be ubiquitinylated by E3-ubiquitin-ligase PUB4, which leads to their rapid degradation. FtsH, protease. Solid arrows indicate confirmed interactions, dotted arrows indicate hypothetic interactions.

**Figure 6:**
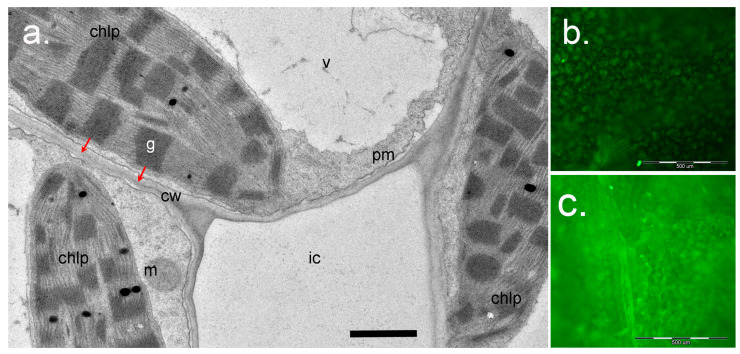
Application of Singlet Oxygen Sensor Green (SOSG) for study of ^1^O_2_ in plants. (**a**), a cross-section of the leaf mesophyll of *Paspalum vaginatum* (Poaceae) (courtesy of Dr. Nouria K. Koteyeva, Komarov Botanical Institute RAS, Saint Petersburg). Positions of chloroplasts (chlp) relative to cell wall (cw) and intercellular space (ic) are shown. Red arrows show the distance for ^1^O_2_ diffusion from the grana (g) to react with SOSG in the water phase of the apoplast in cell walls after infiltration of SOSG solution into the intercellular space. Scale bar: 1 µm. m, mitochondrion; pm, plasma membrane; v, vacuole. (**b**), (**c**)—Visualization of SOSG fluorescence using epifluorescence microscopy. Detached leaves of *Arabidopsis* wild-type Col-0 (**b**) or the *ch*1-3 mutant (**c**) were infiltrated with SOSG (5 µM) and exposed to light (PPFD 233 µmol m^−2^s^−1^) for 2 h. The leaves were then photographed using an Olympus BX51 microscope (Olympus Optical Co., Germany) equipped with a ColorView II digital camera and Cell^F software (Olympus, Hamburg, Germany), with an exposure time of 500 ms, using blue excitation filter (BP 460-490, DM 505, BA 510-550). Scale bar: 500 µm.

Altogether, signaling pathways initiated by ^1^O_2_ and mediated by EX1/EX2 differ from those mediated by OXI1 or β-cyclocitral, respectively (Figure 5). These differences are probably based on the short lifetime and high reactivity of ^1^O_2_. It is assumed that ^1^O_2_ cannot diffuse over a distance longer than a half of grana length (approx. 250 nm; Figure 6). Thus, ^1^O_2_ production at grana margins probably does not influence the oxidation of β-carotene in PSII reaction centers located in grana cores, while ^1^O_2_ produced at grana cores does not reach grana margins to induce EX1 degradation. Recently, a novel ^1^O_2_-activated signaling pathway has been revealed which recognizes damaged chloroplasts and leads to their ubiquitinylation and rapid degradation [99]. A cytoplasmic E3-ubiquitin-ligase PUB4 (plant U-box 4) “marks” chloroplasts with enhanced levels of ^1^O_2_ production, ensuring maintenance of the population of physiologically active chloroplasts in the mesophyll [100,101] (Figure 5). These data were obtained using *Arabidopsis fc2* mutants with defects in plastidial ferrochelatase 2. These plants accumulated high levels of protoporphyrin IX leading to the generation of ^1^O_2_ in the light [99]. Interestingly, there was no activation of EX1-dependent signaling, although it can be assumed that the accumulation of protoporphyrin IX, and therefore the generation of ^1^O_2_, in *fc2* plants occurs close to the complex containing Mg^2+^-protoporphyrin IX monomethyl-ester-cyclase, i.e., at grana margins. Notably, PUB4-mediated degradation of chloroplasts occurs much faster than chlorophagy [101].

Importantly, the local production of ^1^O_2_ in one of the leaves, or in a part of a leaf, of mature *Arabidopsis* plants induces the systemic acclimation to stress in the whole plant [102]. Thus, the influence of ^1^O_2_ on plants extends far beyond the chloroplasts where it is generated.

## 8. Role of ^1^O_2_ in Development of PCD in Plants

As already discussed above, ^1^O_2_ fulfils at least two signaling roles in plants: in the acclimation to excess light conditions, and in the induction of PCD [103]. While the first role is specific to leaves, the second role has been detected also in roots of *Arabidopsis thaliana* [59]. Here, ^1^O_2_-induced PCD of apical meristems might cause an increase in lateral root formation, which is a common response to many abiotic stresses experienced by roots [59]. It can be speculated that in leaves, ^1^O_2_-triggered OXI1-dependent PCD might represent the outcome of an unsuccessful adaptive response, while ^1^O_2_-triggered EX1-dependent PCD might provide an emergency pathway for elimination (often leading to the formation of microlesions on leaves) of leaf cells accumulating highly dangerous chlorophyll precursors/metabolites in an uncontrolled fashion [103,104]. An important but probably underestimated, and clearly not sufficiently studied, function of ^1^O_2_-induced PCD might be the participation in the hypersensitive response during pathogen attack [103,105]. ^1^O_2_-triggered PCD has been described also in photosynthesizing suspension cell cultures of *Arabidopsis* [106]. Altogether, more research is necessary to understand the physiological roles of ^1^O_2_-induced PCD in plants.

Neither the exact source of ^1^O_2_ nor the components of the signal transduction pathway involved in ^1^O_2_-triggered PCD induction are known for roots. The best characterized ^1^O_2_ signaling pathways in leaves are mediated by OXI1 and EX1, respectively; recently, a novel route involving the SAFE1 protein was suggested [91]. Notably, while EX1 and SAFE1 are chloroplastic proteins, OXI1 kinase is expressed not only in leaves but also in roots and plays a role in root hair growth and in the oxidative burst [107]. Transduction of ^1^O_2_ signals leading to the induction of PCD involves JA and SA: synthesis of both groups of phytohormones increases in the course or OXI1- or EX1-mediated PCD, respectively. JA plays a key role in the ^1^O_2_-signaling cascades leading to PCD in both *flu* and *ch*1 mutants, and the choice between PCD and acclimation to ^1^O_2_ also depends on this phytohormone [108]. Interestingly, 12-oxophytodienoic acid (OPDA), the precursor of JA, has an antagonistic effect on PCD initiated by JA in *Arabidopsis* suspension cell cultures [106]. ^1^O_2_-induced PCD requires the synthesis of SA in both *flu* and *ch*1 mutants [88,109]. However, addition of the photosensitizer Rose Bengal to photosynthesizing *Arabidopsis* suspension cell cultures during moderate light stress elicited JA- and ethylene-dependent PCD while the EDS1 protein was not involved, suggesting that SA signaling was not required [106]. Ethylene signaling was also suggested to take part in the ^1^O_2_-mediated PCD in leaves [106,108]. 

It should be noted that there is evidence for cross-talk between ^1^O_2_-signaling and signaling networks related to other ROS: for instance, reduced ROS antagonize ^1^O_2_-mediated signaling which eventually can lead to the delay of PCD [110]. Sabater and Martin [111] hypothesized that the ratios between different ROS might be translated into changes in the JA/H_2_O_2_ ratio, and thus could influence the choice between the activation of the defense response vs. PCD response.

Cytological characteristics of ^1^O_2_-mediated PCD were investigated using plants with modified levels of protochlorophyllide oxidoreductase C, namely *porC*-2 (mutant lacking PORC and overaccumulating Pchlide) and *PORCx* (plants overexpressing *porC*-2) [112]. This study allowed minimization of the production of ROS other than ^1^O_2_ by using low levels of light. Treatment with 5-aminolevulinic acid caused blebbing of the plasma membrane and DNA breaks (revealed by TUNEL-positive nuclei), both hallmarks of apoptosis, in protoplasts of wild-type and *porC*-2 plants [112]. While the low amounts of ^1^O_2_ produced in *PORCx* protoplasts under experimental conditions were not sufficient to initiate PCD, after prolonged exposure to light, PCD occurred also in *PORCx* protoplasts [112].

Interestingly, an increase in ^1^O_2_ production did not lead to cell death in the *Arabidopsis npq*1*lut*2 double mutant lacking the LHC carotenoids zeaxanthin and lutein [113]. At the same time, the ABA deficient mutant *aba*1 lacking zeaxanthin deepoxidase and deficient in two other LHC carotenoids, violaxanthin and neoxanthin, was shown to produce ^1^O_2_ leading to cell death under high light [110]. During ^1^O_2_-mediated cell death in *aba*1 plants, chloroplasts aggregated at the center of the cells; such aggregation is regarded as a step preceding their autophagic degradation [110]. Disruption of chloroplasts was also detected [110].

Thus, light and chloroplasts are of high significance for ^1^O_2_-mediated PCD in leaves: First, ^1^O_2_ is mainly produced in chloroplasts in the light; second, such key regulators of ^1^O_2_-signaling pathways as EX1 and SAFE1 are located in chloroplasts; third, the phytohormones JA and SA regulating ^1^O_2_-mediated PCD are synthesized in chlotoplasts. Nevertheless, PCD is a whole-cell process involving other cell compartments. For instance, it was shown that during pathogen treatment, the red-chlorophyll-catabolite can move from chloroplasts to mitochondria and induce ^1^O_2_-dependent PCD in mutants lacking the ACCELERATED CELL DEATH2 protein [105]. A recent study using the *ceh*1 mutant impaired in isoprenoid synthesis revealed another important player of ^1^O_2_-mediated PCD, the endoplasmic reticulum (ER)-mediated unfolded protein response (UPR) [109]. The UPR is a stress reaction activated in response to the accumulation of aggregates of unfolded and misfolded proteins inside the ER lumen (ER stress) which aims at the restoration of normal ER functions but can elicit PCD if the adaptation was unsuccessful. The role of the URP in ^1^O_2_-mediated PCD was further confirmed using *ch*1 mutants [109]. In *ch*1, expression of UPR genes was found to be constitutively enhanced compared to the situation in wild type plants, and increased further under high light conditions, eventually leading to cell death. Thus, a moderate UPR served as part of the acclimatory response of *ch*1 plants to ^1^O_2_, and a strong activation of the whole UPR was associated with cell death [109]. Notably, some of UPR-related genes induced in *ch*1 were also induced in the *flu* mutant [109]. It has been shown that URP in plants is regulated by SA [114]. The exact mechanisms of interaction of chloroplasts and ER during ^1^O_2_-mediated PCD remain unknown, but it is proposed that the involvement of the ER in ^1^O_2_ signaling might be mediated via direct contacts between ER and chloroplasts. Chloroplasts are known to form fine protrusions of stroma, called stromules, into the cytoplasm, especially under high levels of ROS production [115]. The ER also has specific membrane extensions, which might connect to stromules [109].

A recent study showed that ^1^O_2_ produced in the vacuole or at the plasma membrane can be involved in vacuole-mediated PCD [116]. In leaves of *Arabidopsis*, ^1^O_2_ generation was induced either by Rose Bengal or by Acridine Orange, which localized to the plasma membrane or to the vacuole, respectively. Photoactivation of both photosensitizers led to induction of PCD of the vacuolar type, but the patterns were different. Disruption of the tonoplast was observed only when ^1^O_2_ was generated by vacuolar Acridine Orange. This process was accompanied by a release of RESPONSIVE TO DESSICATION-21 (RD21) protease into the cytoplasm, its binding to the protective protein AtSerpin and strong acceleration of PCD [116]. Although these data were obtained with artificial photosensitizers, the same study showed that a similar mechanism can operate in leaf cells during acute water stress [116].

Hence, the initiation of PCD by ^1^O_2_ evolved from chloroplasts, as well as from other cellular compartments, both in leaves and in non-photosynthetic plant organs is a highly intricate and not yet understood process. A picture is emerging that there are several signaling pathways transducing the ^1^O_2_-triggered PCD signal into the nucleus, which can interact and compete. Our current understanding of molecular and cytological mechanisms of ^1^O_2_-mediated PCD remains incomplete.

## 9. Detection of ^1^O_2_ in Plants

^1^O_2_ was discovered by Hans Kautsky [117] who noticed that chlorophyll fluorescence in living cells was lower than in chlorophyll extracts and was rapidly quenched. The reason was in vivo quenching of chlorophyll fluorescence by oxygen accompanied by energy transfer and formation of ^1^O_2_ [118]. As the lifetime of ^1^O_2_ and its diffusion distance are very short, the specific tracers and quenchers used for ^1^O_2_ detection should be able to access the sites of ^1^O_2_ generation within the cells as closely as possible [77].

The sites and the levels of ^1^O_2_ production in plant cells can be revealed on the basis of specific ^1^O_2_ reaction products (for instance, lipid peroxidation products) or using specific quenchers like sodium azide or histidine. However, these methods are indirect and the results are equivocal [46]. Direct detection of ^1^O_2_ as well as the estimation of its concentration and time dynamics in a biological environment is a complicated task [119]. Without reactions, ^1^O_2_ returns to its ground state by emission of light of about 1270 nm. Unfortunately, the 1268 nm peak is positioned on the declining spectral component of chlorophyll phosphorescence which makes any quantification impossible [31].

The most selective method of ^1^O_2_ detection is electron paramagnetic resonance (EPR) spectroscopy. EPR spectroscopy detects free radicals; therefore, identification of ^1^O_2_ is based on spectral characteristics of special “trapping” molecules forming radicals after interaction with ^1^O_2_. In 1976, TEMP (2,2,6,6-tetramethylpiperidine) was proposed as a trap for ^1^O_2_ detection [120]. As a strong electrophile species, ^1^O_2_ can oxidize TEMP to its stable N-oxyl radical TEMPO (2,2,6,6-tetramethylpiperidine-1-oxyl) which can be detected with EPR spectroscopy [31,38]. Unfortunately, this technique cannot be used for ^1^O_2_ detection in plants in vivo due to the high sensitivity of nitroxide-radicals to reductants, especially those produced in illuminated thylakoids. Moreover, the relatively high water contents in leaves also interfere with EPR-based methods [33].

To date, a very convenient way to detect sites and levels of ^1^O_2_ generation in plants is provided by specific fluorescent probes which change their properties after reaction with ^1^O_2_ [121,122]. In leaves, ^1^O_2_ generation in chloroplasts in the light can be traced using a fluorescing spin probe DanePy which exhibits bright fluorescence in the absence of ^1^O_2_ but becomes progressively quenched by ^1^O_2_ in a quantitative manner. Experiments with DanePy using photoinhibited isolated thylakoid membranes showed that the light-induced quenching of DanePy fluorescence is specific to ^1^O_2_ [33]. Another commercially available probe is Singlet Oxygen Sensor Green (SOSG) which is highly selective for ^1^O_2_ and does not change fluorescence upon interaction with O_2_^•−^ or OH^•^ [123]. In the absence of ^1^O_2_ SOSG displays a weak blue fluorescence with excitation peaks at 372 nm and 393 nm, and emission peaks at 395 nm and 416 nm. In the presence of ^1^O_2_ SOSG shows bright green fluorescence with excitation and emission peaks at 504 nm and 525 nm, respectively [27]. Reaction of SOSG with ^1^O_2_ leads to formation of SOSG endoperoxide. Intramolecular transfer of electrons quenches the fluorescence of the SOSG chromophore before reaction with ^1^O_2_ while formation of the endoperoxide prevents electron transfer, and SOSG fluorescence can be observed [123].

A drawback of SOSG is its ability to function as photosensitizer after illumination with UV light or after prolonged exposure to high light, thus promoting ^1^O_2_ formation [124]. However, even under these conditions the levels of SOSG fluorescence caused by photosensitization effects are much lower than the levels of ^1^O_2_ production in plants in vivo [94]. This, as well as the high specificity for ^1^O_2_ [123], sensitivity and convenient detection methods (in contrast to DanePy whose fluorescence is quenched by ^1^O_2_, SOSG fluorescence increases proportional to ^1^O_2_ levels) explain the increasing use of SOSG.

Usually, a buffered SOSG solution with a final concentration of 5–100 µM is introduced into leaves or seedlings by means of vacuum-infiltration [94,125]. SOSG was described as not being able to penetrate cell membranes [126]; however, other authors reported that in high concentrations, SOSG can enter cells and accumulate in the nucleus [121,122]. Nevertheless, infiltration of low concentrations of SOSG in leaves, i.e., into the leaf intercellular space where it comes in contact with the apoplastic water phase, seems to represent a convenient method to study ^1^O_2_ generation in chloroplasts. It should be taken into account that in mesophyll cells, chloroplasts are situated very close to plasma membrane and cell walls: the diffusion distance for ^1^O_2_ from chloroplasts to SOSG in the apoplast is about 200 nm (Figure 6). The lifetime of ROS in the apoplast is much longer than inside cells due to the absence of most antioxidant systems [74]. Accumulation of fluorescent SOSG in infiltrated leaves in the light allows estimation of the levels of ^1^O_2_ production and performance of quantitative and semi-quantitative evaluations (see, for instance, [50,76,124]).

Another tool used for studies of ^1^O_2_ production in plants are non-fluorescent quenchers like DABCO (1,4-diazabicyclo[2.2.2]octane, or triethylenediamine TEDA) [127,128] and 2,2,5,7,8-pentamethyl-6-chromanol (PMC) [129]. DABCO is a nucleophilic aliphatic quaternary amine often used in mounting kits for fluorescent microscopy as an antifading agent. PMC is a lipophilic analog of α-tocopherol able to quench ^1^O_2_ and lipid peroxyl radicals [130,131]. Both DABCO and PMC are highly effective ^1^O_2_ quenchers [132]. A disadvantage of DABCO is that its cross-reactivity with other ROS is not fully understood, while PMC is not very specific to ^1^O_2_ and reacts also with superoxide radical [133], ozone [134] and NO [135]. Nevertheless, DABCO and PMC were used in studies of the role of ^1^O_2_ during salt stress-induced PCD in tobacco suspension cells and in the halophyte *Cakile maritima* [127,128].

A very convenient and sensitive method of ^1^O_2_ detection is based on in vivo imaging of whole plants for the detection of autoluminescence. Autoluminescence arises due to spontaneous photon emission (SPE) during decomposition of lipid hydroperoxides and endoperoxides produced via lipid oxidation by ^1^O_2_ or triplet carbonyls [136,137,138,139]. SPE occurs at an intensity several orders lower than that of bioluminescence and has a long relaxation period of up to several hours. The equipment for detection of autoluminescence includes a high-sensitivity cooled CCD camera, a 50-mm focal lens and a set of color filters for the analysis of the spectral characteristics of the autoluminescence [139]. Autoluminescence imaging allows non-invasive quantitation of early symptoms of oxidative stress in plants which can be detected much earlier than visual signs such as chlorophyll bleaching or turgor loss (e.g., [75,76,94,95]).

## 10. Conclusions

Photochemistry and photophysics of ^1^O_2_ constantly and significantly influence plants’ lives. Along with other ROS, ^1^O_2_ is a central component of plant response to stress, and its levels and sites of production can determine the type of response: acclimation vs. programmed cell death. Studies of mechanisms, sites and regulation of ^1^O_2_ formation in plants, as well as understanding of signaling pathways activated by ^1^O_2_, is of high importance for basic and applied research; for instance, results of such studies allowed the development of effective herbicides [10]. Now, it is important to extend the knowledge obtained using *Arabidopsis* to other plant species including crops. The levels of ^1^O_2_ generation affect plants’ adaptation to environmental factors, most prominently to two of them, light and biotic stress induced by pathogens. Studies of interactions between signaling pathways regulated by ^1^O_2_ and by photoreceptors, respectively, as was shown for CRY1 in *Arabidopsis*, might reveal new aspects of the role of ^1^O_2_ in plant acclimation to light conditions, and allow optimization of light conditions in greenhouses.
